# Type 1 Diabetes in a Pediatric Patient With Beckwith-Wiedemann Syndrome

**DOI:** 10.1210/jcemcr/luae122

**Published:** 2024-07-18

**Authors:** Lubaina Ehsan, Reem Anz, Hannah Asebes, Nikoli Nickson, Berrin Ergun-Longmire

**Affiliations:** Department of Pediatrics and Adolescent Medicine, Western Michigan University Homer Stryker M.D. School of Medicine, Kalamazoo, MI 49007, USA; Department of Pediatrics and Adolescent Medicine, Western Michigan University Homer Stryker M.D. School of Medicine, Kalamazoo, MI 49007, USA; Department of Pediatrics and Adolescent Medicine, Western Michigan University Homer Stryker M.D. School of Medicine, Kalamazoo, MI 49007, USA; Western Michigan University Homer Stryker M.D. School of Medicine, Kalamazoo, MI 49007, USA; Department of Pediatrics and Adolescent Medicine, Western Michigan University Homer Stryker M.D. School of Medicine, Kalamazoo, MI 49007, USA

**Keywords:** type 1 diabetes, Beckwith-Wiedemann syndrome, autoimmunity, genetics

## Abstract

Beckwith-Wiedemann syndrome (BWS) is a genetic overgrowth syndrome with multiple clinical manifestations, including hypoglycemia. Various genetic alterations leading to BWS have been described. Literature has also described the association between BWS and congenital diabetes, but little is known about the association with type 1 diabetes (T1D). We report a 4-year-old female patient with co-occurring BWS and T1D. The patient presented with 2.4-kilogram weight loss in 3 months accompanied by headache, polyuria, and polydipsia. Initial workup showed blood glucose of 681 mg/dL (37.8 mmol/L). Additional workup revealed marked elevation of the glutamic acid decarboxylase 65 and insulin antibodies, confirming the diagnosis of T1D. The patient's initial genetic test results revealed BWS caused by hypomethylation of the imprinting center 2 (IC2) found on maternal chromosome 11. Concurrence of BWS and T1D is rare and there are cases previously described where BWS has co-occurred with congenital diabetes but not T1D. Although the etiology of acquired autoimmunity is unclear, the answer may lie in genetic analysis or autoimmunity secondary to preceding viral illness. Regardless of the etiology, this case emphasizes further exploration of the association between BWS and T1D.

## Introduction

Beckwith-Wiedemann syndrome (BWS) is a well-known overgrowth syndrome characterized by various clinical manifestations. One of the metabolic abnormalities frequently observed in BWS is hypoglycemia, affecting up to 50% of diagnosed infants [1, 2]. Literature has mentioned that this hypoglycemia can possibly be secondary to islet cell hyperplasia with corresponding hyperinsulinemia [3]. Despite hypoglycemia being a frequent component of BWS presentation, previous reports have characterized a paradoxical co-occurrence of BWS and congenital diabetes, also known as neonatal diabetes [4, 5].

The genetics of BWS are known to be complex, involving various molecular mechanisms such as hypomethylation of imprinting center 2 (IC2) on maternal chromosome 11, hypermethylation of imprinting center 1 (IC1) on maternal chromosome 11, mutation of maternal *CDKN1C* allele, complete or partial uniparental disomy of chromosome 11, paternal duplications of 11p, and other genetic mutations [6, 7]. Somatic mosaicism has also been observed, further contributing to the phenotypic variability associated with BWS [6, 7]. For diabetes diagnosed among patients with BWS, co-occurrence of loss of methylation at imprinted loci of chromosomes 6q24 and 11p15.5 has been reported [5]. In addition, overexpression of the imprinted genes *PLAGL1* and *HYMAI* on human chromosome 6q24 has been reported to cause transient congenital diabetes mellitus in patients with BWS [8].

A subset of BWS patients with hypomethylation at the *KCNQ1OT1* imprinting control regions (IC2 defect) have also been noted to have loss of methylation at other imprinted loci; hypomethylation of multiple maternally methylated imprinting control regions, including *KCNQ1OT1* has also been demonstrated in cases of transient congenital diabetes [9].

While previous case reports and studies have described the co-occurrence of BWS and congenital diabetes, this report presents a patient with concurrent BWS and T1D.

## Case Presentation

A 4-year-old female with BWS presented to her primary care physician for weight loss accompanied by headaches, polyuria, and polydipsia. A month prior to her presentation, the patient had an upper respiratory tract illness, presumably due to a viral infection. It was noticed that the patient had lost 2.4 kilograms in 3 months with a drop in weight-for-age percentile from the 95th percentile to the 75th percentile. In addition to the weight loss, her parents noticed increased thirst along with increased urination and nocturnal enuresis. The patient was otherwise toilet-trained, thus the nocturnal enuresis was unusual. The patient did not have any concerns regarding bowel movements, nausea, or vomiting. At the time of presentation, her vital signs were within normal limits for her age and the physical examination was unremarkable.

The patient's past medical history included preterm birth at 30 weeks gestational age with a birth weight of 1.31 kilograms. She was twin B in a monozygotic twin gestation. Prenatally, ultrasound confirmed a di-di twin gestation and identified a small omphalocele in twin B. In utero, there was discordant fetal growth, with our twin's fetus 20% larger than twin A. At birth, the patient had mild respiratory distress syndrome, requiring nasal continuous airway pressure (CPAP) for 42 hours. In addition to respiratory distress, the patient presented with mild macroglossia, a small omphalocele (eventually surgically corrected), and a patent ductus arteriosus (requiring ibuprofen for size reduction). The patient's presentation at birth, particularly omphalocele and macroglossia, prompted a genetic workup for hypomethylation epimutations for BWS and Russell-Silver syndrome. Molecular analysis showed hypomethylation of maternal chromosome 11 at imprinting center 2 (IC2), a finding consistent with BWS. During her newborn period, she did not have hypoglycemia. Although the patient's twin sister initially had BWS testing that revealed a lower-than-normal hypomethylation of chromosome 11, it was not enough to diagnose BWS; however, repeat genetic testing confirmed the diagnosis of BWS in the patient's twin sister.

The family history was reportedly negative for congenital anomalies, intellectual disability, multiple miscarriages, or other medical or genetic conditions. There was no known consanguinity.

## Diagnostic Assessment

Given the initial presentation of polyuria and polydipsia, laboratory workup was done. It revealed elevated serum random glucose level of 681 mg/dL (7.8 mmol/L, normal reference range: 70-99 mg/dL; 3.9-5.5 mmol/L), hemoglobin A1C of 12.4% (normal reference range: 4.4-5.6%). Serum bicarbonate was mildly low at 22 mEq/L (22 mmol/L; normal reference range of 23-32 mmol/L/mEq/L). The patient's anion gap, serum creatinine, aspartate transaminase, and alanine transaminase were within normal limits.

In addition to the above, screening labs for celiac disease and thyroid disorder showed mildly elevated transglutaminase IgA at 16 U/mL (normal range: < 15.0 U/mL) and normal thyroid function with negative thyroid autoantibodies. C-peptide was low at 0.9 ng/mL (0.3 nmol/L, normal range: 1.4-4.4 ng/mL; 0.46-1.46 nmol/L). Autoimmune markers of T1D were obtained within 6 months of insulin treatment initiation (Table 1). The tests for T1D autoimmune markers, including zinc transporter 8 (ZnT8) antibody and islet-antigen 2 (IA2) antibody, were sent out and the sample was inadequate. Given that it was a send-out laboratory, the team learned about the sample being inadequate a few days after insulin was initiated. The patient had elevated glutamic acid decarboxylase 65 (GAD65) and insulin autoantibodies (IAA) at 444 nmol/L and 0.44 nmol/L, respectively (normal ranges: ≤ 0.02 nmol/L and ≤ 0.02 nmol/L, respectively). The patient did not have ZnT8 antibody elevation (normal range: < 15.0 U/mL) although she had a mild elevation of IA2 antibodies at 0.06 nmol/L (normal ranges: ≤ 0.02 nmol/L).

**Table 1.** Diabetes autoantibody panel*^a^*

Serum antibody	Normal range	Value
Glutamic acid decarboxylase 65 antibody	≤0.02 nmol/L	**444 nmol/L**
Insulin autoantibody	≤0.02 nmol/L	**0.44 nmol/L**
Islet-antigen 2 (IA2) antibody	≤0.02 nmol/L	**0.06 nmol/L**
Zinc transporter 8 (ZnT8) antibody	<15.0 U/mL	<15.0 U/mL

^*a*^Obtained within 6 months after the patient was started on subcutaneous insulin. Abnormal values are shown in bold font.

With all clinical evidence considered, the patient was diagnosed with T1D without ketoacidosis. The following laboratory tests were obtained via Mayo Clinic laboratories: transglutaminase IgA, GAD65 antibody, IA2 antibody, IAA, and ZnT8 antibody.

## Treatment

The patient was started on a subcutaneous basal and bolus insulin regimen and eventually discharged once lack of urine ketones were detected via point-of-care testing.

## Outcome and Follow-Up

The patient tolerated the subcutaneous basal and bolus insulin regimen, received appropriate diabetic education, and was scheduled to follow up as an outpatient with endocrinology.

## Discussion

Our case report highlights the co-occurrence of BWS and autoimmune T1D, a presentation not yet described in the literature to the best of our knowledge. While the diagnosis in this patient was confirmed, the underlying pathophysiology was not, underscoring the need for further exploration into any association between these 2 conditions. This case also affirms that marked hyperglycemia in patients with an overgrowth syndrome like BWS warrants consideration of co-occurring diabetes, as this case and others before documented the paradoxical presentation of both conditions in the same patient.

In our case report, we present a patient with BWS who was diagnosed with T1D at the age of 4 years. This finding differs from a previously reported case from Europe, in which a 17-year-old boy with BWS was diagnosed with permanent congenital diabetes mellitus at 4 months of age [4]. Importantly, this individual shared the same genetic defect as our patient, namely, the loss of IC2 methylation at 11p15, which was suggested to be the contributing factor to both the BWS diagnosis and the presence of diabetes [4]. Congenital diabetes can be either transient or permanent; patients are usually diagnosed within the first 6 months of life and often have a monogenic defect causing the pathophysiology [10]. Similarly, hypomethylation of loci on chromosomes 6 and 11 has been connected in the co-occurrence of BWS and congenital diabetes [5]. Although our patient demonstrated hypomethylation of IC2 on maternal chromosome 11 and therefore presented with the sequelae of BWS, she did not present with congenital diabetes. Instead, she acquired the condition through autoimmunity years later. While these presentations are similar, there appears to be a co-occurrence of BWS and diabetes in one form or another.

In the case of our patient, the clinical diagnosis of T1D was supported by the presence of GAD65 antibodies in the context of prominent hyperglycemia and associated symptoms. In considering the cause of acquired autoimmunity, genetics may once again prove a link between these conditions; *CDKN1C*, a cell cycle regulator, is a paternally imprinted gene involved in regulating β-cell proliferation and is known to be associated with growth disorders, including BWS and IMAGe (intrauterine growth restriction [IUGR], metaphyseal dysplasia, adrenal hypoplasia congenita, and genitourinary abnormalities) syndrome [11]. Changes in cell cycle regulators may lead to hypofunctioning of β-cells in a way that drives autoimmunity and the development of diabetes. Impairments in the *CDKN1C* gene have also been linked with the development of early-adulthood-onset diabetes and may provide a linkage between these 2 factors: IC2 hypomethylation at chromosome 11, as described in our patient, affects the expression of *CDKN1C* gene [11]. Other than genetic linkage, another potential etiology is autoimmunity secondary to viral infection, which our patient had 1 month prior to presentation; enterovirus infections have been implicated as potential environmental triggers for the development of autoimmune diabetes in susceptible individuals [12]. While not a frequent occurrence for such a common infectious disease, it is a possibility that this was the driving mechanism for the acquired diabetes. Of note, IA2 antibodies were mildly elevated in our patient. Since the repeat sample for these was obtained almost 6 months after insulin initiation, it may be a depiction of the underlying autoimmunity, although that remains unclear.

BWS is a condition estimated to occur in 1 in 11 000 births [13] while T1D is estimated to occur in 1.93 per 1000 children under the age of 20 [14]. In only the consideration of these incident rates independent of linking factors, the anticipated rate of co-occurrence would be 1 in approximately 5 700 000 cases. The presence of factors linking these 2 conditions together, such as genetic mechanisms, would increase the incident rate more than what is calculated. Further studies are required to reveal more clear linkages between the 2 conditions that would suggest a greater rate of co-occurrence than anticipated.

## Learning Points

- Beckwith-Wiedemann syndrome (BWS) is a genetic condition that often presents with hypoglycemia, and existing literature demonstrates the co-occurrence of BWS and congenital diabetes but not type 1 diabetes (T1D).
- Our case report presents a novel insight of T1D being a concurrent condition in patients with BWS.
- There may be a shared genetic linkage between cell cycle regulator genes for β-cells that could be affected in BWS, driving autoimmunity and the development of diabetes.

## Contributors

L.E., R.A., H.A., B.E.L.: Involved in the diagnosis and management of this patient.

N.N.: Involved in manuscript writing and editing.

B.E.L.: Responsible for the patient's management and follow-up.

L.E., R.A., H.A., N.N., B.E.L.: Also involved in manuscript writing, editing, and review.

## Data Availability

Data sharing is not applicable to this article as no datasets were generated or analyzed during the current study.

## Abbreviations

BWS: Beckwith-Wiedemann syndrome
GAD65: glutamic acid decarboxylase 65-kilodalton isoform
IA2: islet-antigen 2
IAA: anti-insulin autoantibodies
IC2: imprinting center 2
T1D: type 1 diabetes
ZnT8: zinc transporter 8
